# Rheumatic? A diagnostic decision support tool for individuals suspecting rheumatic diseases: Mixed-methods usability and acceptability study

**DOI:** 10.1186/s41927-025-00507-w

**Published:** 2025-05-23

**Authors:** Stefan Jakobi, Katharina Boy, Magali Wagner, Susann May, Alp Temiz, Anna-Maria Liphardt, Elizabeth Araujo, Loreto Carmona, Rachel Knevel, Georg Schett, Johannes Knitza, Felix Muehlensiepen, Harriet Morf

**Affiliations:** 1 Department of Internal Medicine 3 - Rheumatology & Immunology, Universitätsklinikum Erlangen Friedrich-Alexander-Universität Erlangen-Nürnberg, Ulmenweg 18, 91054 Erlangen, Germany; 2 Deutsches Zentrum für Immuntherapie (DZI), Friedrich-Alexander-Universität Erlangen-Nürnberg and Uniklinikum Erlangen, Erlangen, Germany; 3 Center for Health Services Research, Faculty of Health Sciences Brandenburg, Brandenburg Medical School Theodor Fontane, Seebad 82/83, Rüdersdorf bei Berlin, Germany; 4 Instituto de Salud Musculoesquelética (INMUSC), Madrid, Spain; 5 Department of Rheumatology, Leiden Universitair Medisch Centrum, Leiden, 2333 ZA The Netherlands; 6 Department of Rheumatology, Newcastle Upon Tyne Hospitals NHS Foundation Trust, Newcastle Upon Tyne, NE7 7DN UK; 7 Institute for Digital Medicine, University Hospital of Giessen and Marburg, Philipps University Marburg, Marburg, Germany; 8 AGEIS, Université Grenoble Alpes, Grenoble, France

**Keywords:** Usability, Usability study, eHealth, Telemedicine, Health services research, Arthritis, Symptom checkers, NPS, Triage

## Abstract

**Background:**

The early diagnosis of inflammatory rheumatic diseases (IRDs) is of paramount importance in order to prevent irreversible damage to joints and to optimize treatment outcomes. Nevertheless, conventional care pathways frequently entail diagnostic delays spanning several months. Symptom checkers (SCs) have the potential to provide a solution by offering validated symptom assessments, improving triage systems and expediting diagnostic evaluations. The objective of this mixed-methods study is to assess the usability and acceptability of the SC *Rheumatic?* among individuals with suspected rheumatic diseases.

**Methods:**

A total of 105 individuals with suspected IRDs who were newly presenting at an outpatient rheumatology clinic completed the *Rheumatic?* symptom checker and an evaluation questionnaire. The questionnaire comprised the System Usability Scale (SUS) and Net Promoter Score (NPS). Additionally, 14 participants were interviewed by telephone in order to gain further insights through the qualitative method.

**Results:**

The *Rheumatic?* symptom checker received a “good” usability score, with an average SUS of 78 ± 16 (range 0-100). Younger participants reported significantly higher usability scores (*p* < 0.03). However, the NPS was − 15 (range − 100 to 100), indicating lower acceptability. Qualitative data supported the positive usability ratings but emphasized the need for enhancements to increase user engagement and perceived value, such as a current perceived lack of immediate benefit for many users. Their experience varied in terms of impact, with some patients suggesting an increased awareness of their symptoms while others did not notice any difference.

**Conclusion:**

*Rheumatic?* demonstrates good usability, particularly among younger users. Interviews revealed valuable suggestions for improvements, which could enhance overall acceptability and user satisfaction. Implementing *Rheumatic?* could lead to more efficient triage, potentially reducing diagnostic delays and an optimized allocation of resources. Future iterations should prioritize implementation strategies to maximize user impact and benefit.

**Clinical trial number:**

Not applicable.

**Supplementary Information:**

The online version contains supplementary material available at 10.1186/s41927-025-00507-w.

## Introduction

The early diagnosis and prompt treatment of inflammatory rheumatic diseases (IRDs) are critical to improving long-term patient outcomes and preventing irreversible joint damage [1]. The European League Against Rheumatism (EULAR) recommends to see patients within 6 weeks from symptom onset to ensure a therapy start for at-risk patients within 3 months (“window of opportunity”) from symptom onset [2]. Unfortunately, the typical interval between the onset of symptoms and a definitive diagnosis is between four and 40 months, with considerable variation across countries and healthcare systems [3–5]. Prompt and early diagnosis and subsequent treatment increase the probability of remission and reduce the risk of requiring additional, more intensive drug therapies and furthermore prevents the occurrence of irreversible joint damage, including destruction, erosion and deformities. Preserving musculoskeletal function can improve quality of life, pain and work activity.

This diagnostic delay [6, 7] is complicated by the growing prevalence of IRDs [8], with estimates suggesting a current prevalence of up to 3% in Germany [8]. The global prevalence of rheumatoid arthritis (RA) was 17.6 million in 2020 and is projected to increase by 80% to 31.7 million by 2050 [9]. Based on the estimated ratio of two rheumatologists to 100,000 capita [10, 11], it is evident that there is a shortage of these specialists in numerous Western countries [11]. This shortage is likely to intensify because of the ageing workforce [10, 12]. As indicated by the German Association for Rheumatology (DGRh), the number of clinical full-time equivalent practitioners required to meet this demand is approximately equivalent to the number of rheumatologists currently in practice [10]. 

Misdiagnosis by non-specialist physicians poses another problem, with gout and undifferentiated arthritis being common misdiagnoses for first-time rheumatology patients​ [13]. Notably, around 60% of patients presenting to rheumatology clinics with suspected IRDs do not end up receiving an IRD diagnosis [14, 15]. Consequently, patients who do not have a rheumatic disease consume time and medical capacity of the few rheumatologists for themselves. This situation could be further exacerbated by the increase in musculoskeletal complaints within an aging population [16, 17]. 

Despite the critical importance of early diagnosis and treatment, the aforementioned circumstances result in a significant gap in the current diagnostic pathways for IRDs. The current system is inefficient in identifying patients with suspected IRDs, as it both delays the identification of at-risk patients and misclassifies non-IRD patients as being at-risk.

To address these challenges, the development of counter measures, such as more efficient triage systems, are imperative. The utilization of digital tools within the field of medicine is rapidly increasing [18–20], with both users [19, 21] and rheumatologists [19] identifying them as valuable adjuncts to the management of diseases. Importantly, individuals are increasingly turning to online resources to assess new symptoms early in their disease journey. Median time from symptom onset to online search was previously reported to be 2 weeks, with requests for physician appointments occurring at 4 weeks, and the first physician appointment happening at 5 weeks [4]. These findings underscore the potential role of digital symptom assessment tools in accelerating the diagnostic process. Symptom checkers (SC) are designed to prompt the user to input their symptoms and then output one or multiple potential diagnoses or further advice for seeking assistance. SCs could improve triage by providing an initial assessment, diagnosis, or recommendation. This could reduce the number of incorrect referrals, freeing up much-needed specialist time. Additionally, it could facilitate faster diagnosis and specialist referral for at-risk patients. Improved triage systems could also differentiate between patients urgently needing specialist care and those who could be managed in primary care, leading to more efficient use of specialists’ time. The usability of SCs is frequently reported to be good [21–22], and they are well accepted by patients [20–22]. In a comparison between an AI-based and a questionnaire-style SC, Knitza et al. demonstrated that patients found them easy to use and would prefer them to the current “gold standard” of conventional online search engines [21]. The completion time for SCs was considerably shorter than for online search engines, suggesting potential time savings with SC use. However, the accuracy of SCs was still limited [14]. Moreover, over 80% of patients stated that they would not have done anything differently if they had used an SC before their consultation [21]. Given this context and the fact that many rheumatic patients are open to using symptom checkers, even though their actual usage is significantly lower, it is crucial to understand the challenges patients face when using symptom SCs and to work on improving these aspects [18]. 

This study aims to investigate usability and user acceptance of the SC *Rheumatic?* [23] with both quantitative and qualitative means in a real-world environment of an outpatient rheumatology clinic.

## Materials and methods

### Rheumatic?

*Rheumatic?* is a free, publicly available SC that was developed in 2019 together in collaboration with rheumatology experts from multiple European hospitals and patients [24]. It was part of the Joint Pain Assessment Scoring Tool (JPAST) project funded by the European Union, aiming to accelerate diagnosis and early treatment in rheumatology. The version used in the survey was from 2023. Although it does not have a public scoring system and it isn’t certified as a medical product, *Rheumatic?* has demonstrated its capability for the early identification of IRDs in comparison to other musculoskeletal conditions [25]. It has shown high usability and acceptance in a large-scale online survey across all age groups, with the primary patient-perceived limitation being the high number of questions [26]. The tool remains under active development and is incorporated into several ongoing prospective studies.

In the public version utilized for this study (available at https://rheumatic.elsa.science/), users do not receive a result in the form of a diagnosis or care-seeking advice. However, they have the option to download a PDF summary of their symptoms. The questionnaire primarily consists of multiple-choice questions, with some single-choice questions addressing demographics, such as age, sex, alcohol consumption, and smoking status, as well as sliding scale questions for weight and height. Certain questions also include images or graphics. Based on prior responses, additional questions are presented to further explore the symptoms. Users are required to answer a minimum of 17 questions, which encompass demographic and physical attributes, alcohol consumption, and smoking status, with a total of up to 76 questions possible, depending on their symptoms and previous answers. The completion time typically ranges from 5 to 15 min, depending on the number of symptoms reported by the patient. *Rheumatic?* is available in English, Dutch, German, Swedish and Spanish. The German version was used for this study.

### Study design

Patients were recruited consecutively between June 2023 and April 2024 from the outpatient rheumatology clinic at the university hospital Erlangen during their initial visit, until the target sample size of 100 was reached. The patients were selected at random. Due to time constraints and the availability of doctors, patients were only recruited once a week as part of the triage consultation. Eligible patients were adults (18 years or older) with suspected rheumatic diseases, presenting with various musculoskeletal symptoms (e.g. joint pain, muscle pain, stiffness) or prior medical and laboratory test results suggesting a rheumatic disease. Patients were included if they were visiting a rheumatologist for the first time and lacked a prior diagnosis from a specialist. Exclusion criteria were an existing rheumatologic diagnosis or lack of consent. Prior to their consultation with the physician, patients who provided written consent completed both the SC *Rheumatic?* and a usability questionnaire in the waiting room.

### Quantitative analysis

Usability was assessed with the System Usability Scale (SUS) [27] and the Net Promoter Score (NPS) [28]. The SUS comprises 10 statements that respondent’s rate on a five-point Likert Scale, reaching from “strongly disagree” to “strongly agree”. The total score combines all responses to a maximum score of 100. In a recent meta-analysis, the authors conclude that the SUS and the widely accepted benchmark of a mean SUS score of 68 (SD 12.5) are suitable for assessing the usability of digital applications [29]. The NPS is assessed using a single question: “How likely is it that you would recommend [the Symptom Checker *Rheumatic?*] to a colleague or friend?”. This question employs a numeric rating scale (NRS) with a range from 0 to 10. Patients rating 9 or 10 are classified as “promoters”, those rating 7 or 8 as “indifferent” and all others as “detractors”. The final score is then calculated by subtracting the percentage of detractors from the percentage of promoters [28]. The NPS is used in a number of studies to assess the acceptability of digital applications [30–33]. 

Additionally, three non-standardized questions using an NRS and one employing an 11-point Likert Scale were included to assess understandability of the questions and to determine whether patients found the usage of *Rheumatic?* beneficial for clarifying their complaints. Both *Rheumatic?* and the questionnaire were answered on tablet computers. Additional data collected included age, sex, current pain level, final diagnosis, time from symptom onset and current working status.

### Statistical analysis

Statistical analyses were conducted using R version 4.4.1. Continuous variables were summarized as mean ± standard deviation (SD) or median with interquartile range (IQR), depending on their distributional characteristics observed through graphical methods (e.g., histograms, density plots). Welch’s t-test was used for group comparisons, as it accounts for unequal variances, with Hedges’ g reported as the effect size measure. 95% confidence intervals (CI) were provided to indicate the range of plausible effect sizes. Associations between continuous variables were assessed using Pearson’s correlation coefficient (r), with 95% confidence intervals reported to quantify the strength and direction of relationships. Additionally, Bayesian analyses were conducted to assess the strength of evidence for group differences, reporting Bayes Factors (BF) and 95% credible intervals. By integrating both frequentist and Bayesian approaches, this analysis ensures a comprehensive and robust interpretation of the data.

### Qualitative analysis

To explore the patients’ experiences with *Rheumatic?*, we conducted qualitative interviews. The interviews took place between Dezember 2023 and March 2024. A total of 14 participants were selected using purposive sampling [34], a method that allows for the intentional selection of individuals based on predefined criteria to ensure a diverse range of perspectives. The sampling strategy aimed to achieve a heterogeneous sample with regards to age, sex, educational and professional background of the interviewed patients. The initial recruitment of participants was facilitated by our colleagues at the Friedrich-Alexander University Hospital Erlangen. The interviews were conducted via telephone by K.B. using an interview guide that was developed to specifically elicit the participants’ experiences (additional file 2). The semi-structured interview guide consisted of open-end questions that explored the user perspectives towards *Rheumatic?*. The following main topics were investigated: acceptance, benefits and drawbacks, and transferability to standard care. The initial exploratory questions were then refined through follow-up questions. We conducted two pilot interviews to test and refine the interview guide. No revisions were necessary. In addition, socio-demographic data was collected, including gender, age, current occupational status and occupation. The interviews were transcribed and anonymized. Data analysis was conducted by two experienced researchers (K.B. and F.M.) based on Kuckartz’s structured qualitative content analysis [35] using MAXQDA software (Verbi GmbH).

### Ethics approval and consent to participate

The study protocol was approved by the medical faculty ethics committee of the Friedrich-Alexander-Universität Erlangen-Nürnberg, Erlangen, Germany (19-346-B). The study was conducted in accordance with the ethical guidelines of the Declaration of Helsinki. All patients were coded with a consecutive number in a pseudonymization procedure. The collected data was stored and analyzed in a password-protected database that could only be accessed by authorized persons. Patients had the option of withdrawing their participation in the study at any time, whereby all personal data was irrevocably deleted. There was no financial compensation for participating in the study. No trial registration was necessary.

## Results

### Quantitative results

A total of 108 patients were recruited between June 2023 and April 2024. All patients completed both the SC and the questionnaire. In three patients socioeconomic or clinical data was missing. Subsequently 105 patients were included in the statistical analysis, of whom 68 (65%) were female and 37 (35%) were male. The mean age of the participants was 50 ± 16 years. A total of 52 patients (50%) were diagnosed with an inflammatory disease. The mean time from symptom onset was 48 ± 62 months, with multiple patients experiencing symptoms for over 10 or even 20 years, leading to a substantial variability in symptom duration. A total of 76 patients (72%) were still working. Full demographic characteristics are seen in the Table 1 below.

**Table 1:** Clinical and socioeconomic characteristics in a survey of 108 patients (65% female, 35% male) in Erlangen, Germany, from June 2023 to April 2024. IQR, interquartile range; NRS, numeric rating scale (range 0-10); SD, standard deviation; RA, rheumatoid arthritis; spa, spondyloarthritis; PsA

Characteristics	Total (*N* = 105)
Age, *years (IQR)*	50 (26)
Sex, *n (%)*	
- female	68 (65%)
- male	37 (35%)
Pain, *NRS (SD)*	4.49 (2.54)
Diagnosis, *n (%)*	
- RA	17 (16)
- SpA	20 (19)
- PsA	5 (4.8)
- Gout	1 (1.0)
- Osteoarthritis	5 (4.8)
- Sarcoidosis	2 (1.9)
- Fibromyalgia	2 (1.9)
- PMR	2 (1.9)
- Undifferentiated collagenosis	9 (8.6)
- other non-IRD	46 (44)
Time from symptom onset, *months (SD)*	48 (62)
Working status, *n (%)*	
- working	76 (72)
- not working	5 (4.8)
- retired	16 (15)
- on sick leave	8 (7.6)

Characteristics of study population. IQR, interquartile range; NRS, numeric rating scale (range 0–10); SD, standard deviation; RA, rheumatoid arthritis; SpA, spondyloarthritis; PsA, psoriatic arthritis; PMR, polymyalgia rheumatica

**Fig. 1:**
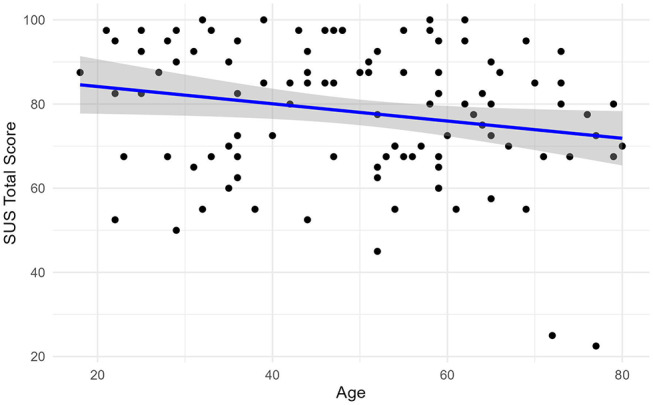
Correlation between age and SUS total score. Correlation between participants age and total SUS score (range 0-100, >68 = “above average” usability, 71.4< x<85.5 = “good” usability) in a survey of 108 patients (65% female, 35% male) in Erlangen, Germany, from June 2023 to April 2024

Total SUS Score (0–100) was 78 ± 16. Older patients rated usability significantly lower than younger patients (*p* < 0.03) with a negative Pearson correlation coefficient of -0.21 (-0.38, -0.02) showing the small but notable effect. Bayesian analysis also showed moderate evidence for the relationship, with a Bayes Factor (log (BF01) =-0.28) supporting the negative association (Fig. 1).

The NPS classified only 26 Patients as promoters of *Rheumatic?*, 35 as indifferent and 43 as detractors (Table 2), resulting in a negative NPS score of -15. The number of questions was evaluated using an eleven-point Likert scale, ranging from “too few questions” to “too many questions”. The mean answer was 5.76 ± 1.99, indicating a good middle ground. The other non-standardized questions had a NRS ranging from 0 to 10. Participants rated the clarity of the questions at 8.48 ± 1.81, their ability to describe their complaints as generally positive at 6.99 ± 2.04, and the overall usefulness of using *Rheumatic?* for clarifying their complaints at 6.34 ± 2.38. The influence of current pain, duration of symptoms, age and diagnosis on the NPS, SUS, and additional questions was examined. RA patients rated the number of questions as excessive (*p* = 0.03) yet perceived them to be clearer (*p* = 0.038). Due to the small sample size of the disease-specific subpopulations, these findings should be interpreted with caution. No other statistically significant correlations were found.

**Table 2:** Results of usability questionnaire in a survey of 108 patients (65% female, 35% male) in Erlangen, Germany, from June 2023 to April 2024. NPS, net promoter score; SD, standard deviation; SUS, system usability scale

Question	Score
NPS Score (0–10), *mean (SD)*	6.72 (2.53)
NPS class, *n (%)*	
- Promoter (Score 9–10)	27 (26)
- Indifferent (Score 7–8)	35 (33)
- Detractor (Score 0–6)	43 (41)
SUS Score (0-100), *mean (SD)*	78 (16)
Usefulness for clarifying complaints (0–10), *mean (SD)*	6.34 (2.38)
Number of questions (0–10), *mean (SD)*	5.76 (1.99)
Clarity of questions (0–10), *mean (SD)*	8.48 (1.81)
Possibility to describe complaints (0–10, *mean (SD)*	6.99 (2.04)

### Qualitative results

#### Participant characteristics

Participant characteristics included diverse occupational backgrounds: One person retired due to medical reasons, one was currently on sick leave, while the others did not currently have any official restrictions or medical leave. All patients were suspected of having a rheumatic disease and had used *Rheumatic?* within the initial consultation. The interviews lasted between 10 and 23 min (mean 21.03).

#### Acceptability and usability

Patients demonstrated overall acceptance of digital healthcare approaches and the use of *Rheumatic?* was a novel component in their healthcare management. The process of completing the digital forms was generally perceived as appropriate and user-friendly. Importantly, patients supported the routine integration of *Rheumatic?* into standard rheumatological care, particularly as a means to optimize waiting periods before initial consultations. Furthermore, *Rheumatic?* contributed to an enhanced patient experience, with many participants reporting a sense of being acknowledged and heard throughout their interactions with the system.

It took me like 15–20 minutes, I had to wait anyhow so this was a pleasant pastime. It was super easy to use– just answer the question and immediately get the next one, very user-friendly. (P 4, Pos. 59–65)

Everything was well explained, clear and you could answer everything right away. If unsure you could just tick ‘I don’t know. (P 9, Pos. 55)

The physician can’t ask everything, but this way they see exactly what you need and how you’re doing. That was good and I felt taken care of. The waiting time was shortened and I felt important. The effort was fine for me. (P 11, Pos. 99–101)

#### Perceived effect of rheumatic?

Patients’ experiences of using *Rheumatic?* varied in terms of its impact. Patients reported that they had a greater awareness of their symptoms and condition after usage, as it encouraged them to think about their health in advance. Others did not notice any significant impact, especially if their physician did not refer to it during the consultation. Patients also described how using *Rheumatic?* made them feel well cared and appreciated that the questions on the website were carefully designed to address their specific concerns, making them feel heard and valued.

Maybe it helped me in that I had to think about it again beforehand more awareness of my symptoms and condition. (P 2, pos. 73)

Actually, no influence, because I can’t remember the physician mentioning it during the conversation. (P 3, pos. 97)

Impact? Well, I felt well taken care of and subsequently felt good that my interests… that someone was interested in how I feel or how I am doing. That was important to me.

Not such lapidary questions, but ones that simply fit my problem well. (P 9, Pos. 83)

Although patients found the questionnaire straightforward and useful, the feedback or the result of *Rheumatic?* was unclear to many.

I don’t think I got any result. (P 4, Pos. 67)

I didn’t knowingly receive any result. (P 5, Pos. 103)

I don’t know if any result came out of it. Did something come out? (P 6, Pos. 59)

Exactly. So, I don’t know how it turned out, this test. (P 10, Pos. 73)

No, I would say it was just a questionnaire. (P 12, Pos. 83)

#### Implementation into the rheumatology care pathway

Patients suggested *Rheumatic?* as a pre-diagnostic tool that could be integrated into standard care to increase efficiency and optimize medical consultations.

I like it as a filter, as a preliminary anamnesis. It could be integrated into the consultation as standard: Like Fill this out - Possibly even at the general practitioners.

This way, you can already see signs of rheumatic disease. If necessary, you could make an appointment with a rheumatologist directly or use it in the clinic to shorten the conversation with the physician. The physician could then ask specific questions and skip other topics, which saves time. (P 6, Pos. 67)

## Discussion

### Principal results

This study demonstrates that the *Rheumatic?* SC is well accepted among rheumatic patients, although it performs rather mediocrely in the NPS score. As expected, user-friendliness is better perceived at a younger age. As suggested by Bangor et al., [36] a score of > 68 indicates an above-average experience, while a SUS score between 71.4 and 85.5 indicates a positive user experience. This places *Rheumatic?* in the “good” range, with a mean value of 78 ± 16 (range 0-100). This positive assessment was further corroborated by patients’ responses during the qualitative interviews, which indicated that the questions posed by *Rheumatic?* were comprehensible and effectively captured the complaints expressed by patients.

Conversely, the NPS yielded less favorable results, with a mean score of 6.72 ± 2.53 (range 0–10). 26% (27/105) of the participants were classified as promoters, 33% (35/105) as indifferent and 41% (43/105) as detractors, resulting in an overall score of -15 (range − 100 to 100). Negative scores are generally interpreted as indicators of low acceptance. In light of the generally positive outcomes, these findings suggest that while users may perceive *Rheumatic?* as a beneficial tool, they are unlikely to actively recommend it to other patients. The interviews also suggest that since patients did not get a diagnosis or advice from *Rheumatic?*, they were unsure about the actual benefit of *Rheumatic?*, which could also lead to a lower NPS. While there is a scoring system for *Rheumatic?*, it is not yet implemented in the public version used in this study [25]. This system provides three potential outcomes: no recommendation, a recommendation to visit a general physician, or a recommendation to visit a rheumatologist. This would make Rheumatic a unique SC for rheumatologic complaints and could thus shorten the diagnosis time. It would also filter out musculoskeletal complaints that do not require a rheumatologist, but only a general physician. Integrating such recommendations as immediate results could improve perceived utility and increase user acceptance and NPS. It should be noted that it is currently discussed how valid the NPS is in countries outside the US [37], where it originated from [28], and in healthcare in general [37, 38]. Cultural differences in rating behavior have been mentioned as a possible cause for this; suggesting Americans are more likely to provide extreme ratings [38, 39] that are necessary for a good NPS.

### Comparison with prior literature

These results are also reflected in recent publications. A Dutch study from 2023 [26] on the user-friendliness of *Rheumatic?* showed that the symptom checker was well accepted by women and men across all ages. Surprisingly, there was a clear difference in the NPS. 74% would recommend the questionnaire to a friend or patient. As noted, before, cultural differences could be the reason for this difference in the NPS scoring but also study design. The surveys did also differ in terms of the questioning and recruiting. In the Dutch study, patients were recruited via the Internet and did not attend a rheumatology clinic at the time of study participation [26]. Our study evaluated *Rheumatic?* in an isolated research context, where patients completed the symptom checker prior to their consultation with the rheumatologist. However, the results were not shared with the rheumatologist, meaning there was no immediate benefit for either the physician or the patient. So far, there are no specific SCs in rheumatology except Rheport. Only self-tests are available on the internet, but these have not been tested scientifically. In another German study from 2022 [21], Knitza et al. compared two different SCs (Ada and Rheport). Ada is an artificial intelligence-driven chatbot app first which asks first for basic health information and then for the current leading symptoms. The total number of questions depends on the previous answers. Ada then suggests five possible diagnoses with probability and risk. The app is not limited to rheumatic diseases. Rheport was developed especially for rheumatic complaints and consists of basic health and rheumatic-specific questions. At the end, the probability of a rheumatic disease is calculated.

In the controlled randomized study, the SUS score of both SCs were comparable to *Rheumatic?* (74 and 77 compared to 78), while patients were more likely to recommend these to friends or other patients (73% and 79% respectively). However, they did not use the NPS for their study, but a simple yes/no question. Nevertheless, the study also showed that the sensitivity and specificity for the detection of rheumatic disease was only around 50% for both SCs.

In our survey, usability was found to be significantly related to age, which is not surprising. A nationwide survey showed that the use of symptom checkers was associated with younger age, higher income and female sex [40]. It is interesting to note that similar effects were found previously with other SCs and similar study designs [21] while the large-scale Dutch study investigating *Rheumatic?* found no associations of usability scores for sex and age [26]. As discussed above there are differences in questioning and study design that could explain the variation.

In the interviews, patients noted the perceived lack of impact, since they did not get an immediate result in form of a diagnosis or advice on how to proceed. This was also a complaint in the Dutch study [26]. The SCs evaluated in the German study did provide the users with such results, however over 80% of patients stated they would not have done anything different after using the SC [21]. 

Both the Dutch [26] and the German [21] study are in line with our results regarding the usability of SCs in the field of rheumatology, indicating that patients with rheumatic complaints approve of the use of symptom checkers, while there is still need to improve them. Another factor to consider is the patients eHealth literacy, meaning the skills and *“the ability to seek*,* find*,* understand*,* and appraise health information from electronic sources and apply the knowledge gained to addressing or solving a health problem”.* [41] While this was not measured in this study, eHealth literacy was reported to be low among rheumatic patients [18, 42]. Although many patients use the Internet to search for health-related topics, there are still many who are unable to use disease-specific applications to their best advantage. Furthermore, symptom checkers do not yet appear to be widespread in Germany and a recent status quo survey [43] revealed that many patients do not see any major benefits, particularly in the field of primary care. The use of symptom checkers was particularly high among older patients (51–55 years) [43], which may be due to chronic illnesses in old age.

### Perspective

Previous studies have already shown that symptom checkers can support the work of doctors [44] and lead to a diagnosis more quickly.

Especially in areas with few specialists, such as rheumatology, the use of symptom checkers may hold high potential to improve patient journeys. So far, however, there is no SC that has been approved and tested for rheumatological complaints.

*Rheumatic?* appears to be a good way of identifying the risk of developing a rheumatic disease.

Further studies should investigate how accurately the symptom checker matches the medical diagnoses in order to be able to integrate its use into the diagnostic process.

However, it must be ensured that the symptom checker is specifically designed for rheumatological complaints and has been developed together with rheumatologists. A recent survey of patients with neurotological complaints who consulted Dr. Google, reported that the medical diagnosis by a neurologist did not match the AI-generated diagnosis [45]. 

### Limitations

The study has several limitations. Firstly, the small number of patients should be mentioned. Although the number is sufficient for static statements, a larger number of patients can make more precise statements about user-friendliness. Also, this was a monocentric study conducted at a university hospital, with referred patients. Future studies should adopt a multi-center approach, incorporating clinics and practices, to increase sample size and enhance representativeness of the study population. Additionally, the idea of the symptom checker could be better explained. Ideally, individuals might also be recruited via the internet or at general physicians. As *Rheumatic?* is available in multiple languages, similar studies should be carried out in different countries to evaluate its usability and acceptability across different healthcare systems.

## Conclusion

The Rheumatic SC demonstrated high usability, especially among younger individuals. However, a low NPS indicated limited acceptance, highlighting the need for further adaptations identified through user interviews. To enhance acceptance, implementation adjustments are recommended, particularly by incorporating result discussions into clinical consultations and linking completion to direct actions.

## Electronic supplementary material

Below is the link to the electronic supplementary material.

Additional file 1: Characteristics of interview participants, description: Table that contains socioeconomic data of the interview participants.

Additional file 2: Interview guide, description: The interview guide used for the qualitative phone interviews in German.

Additional file 3: Interview guide, description: The interview guide used for the qualitative phone interviews in English.

## Data Availability

All data supporting the findings of this study are available within the paper and its additional files. The data sets are available on reasonable request from the corresponding author.

## Abbreviations

IRD: Inflammatory rheumatic disease
SC: Symptom checker
SUS: System usability scale
NPS: Net promoter score
EULAR: European League Against Rheumatism
RA: Rheumatoid arthritis
DGRh: Deutsche Gesellschaft für Rheumatologie
NRS: Numeric rating scale
IQR: Interquartile range
SD: Standard deviation
SpA: Spondyloarthritis
PsA: Psoriatic arthritis
PMR: Polymyalgia rheumatic
US: United States of America
AI: Artificial intelligence
