# Supplementary material for: Rheumatic? A diagnostic decision support tool for individuals suspecting rheumatic diseases: Mixed-methods usability and acceptability study
Source: BMC Rheumatol. 2025 May 23;9:59. doi: 10.1186/s41927-025-00507-w (PMC12101040; doi:10.1186/s41927-025-00507-w)
Supplement: Supplementary file 1 — Additional file 1: Characteristics of interview participants, description: Table that contains socioeconomic data of the interview participants. [file 41927_2025_507_MOESM1_ESM.docx]

Characteristics of interview participants.

| **Patient** | **Age** (years) | **Gender** | **Retired/**  **Unable to work/**  **On sick leave** | **Occupation** | **Education** |
| --- | --- | --- | --- | --- | --- |
| **1** | 29 | male | no | Metal Worker | Secondary School  diploma |
| **2** | 78 | female | no | Pensioner / Medical Assistant | High School Degree |
| **3** | 61 | male | no | IT | Secondary School  diploma |
| **4** | 60 | female | yes | Saleswoman | Secondary School  diploma |
| **5** | 58 | female | no | Therapist | Secondary School  diploma |
| **6** | 63 | male | retired | Value Analyst | Secondary School  diploma |
| **7** | 52 | female | no | Psychology Student / Case Manager /  Freelance Artist | High School Degree |
| **8** | 70 | male | no | Pensioner/ Offset Printer | Vocational diploma |
| **9** | 54 | female | On sick leave | Geriatric Nurse | University Degree |
| **10** | 75 | male | no | Pensioner / Forest engineer | Vocational diploma |
| **11** | 39 | female | no | OR Nurse | Secondary School  diploma |
| **12** | 44 | female | Compensation for disadvantages at school / no | Nurse Student | Secondary School  diploma |
| **13** | 42 | female | no | Office Administrative Staff | Secondary School  diploma |
| **14** | 53 | male | no | Machine adjuster | Secondary School  diploma |
