# Supplementary material for: Rheumatic? A diagnostic decision support tool for individuals suspecting rheumatic diseases: Mixed-methods usability and acceptability study
Source: BMC Rheumatol. 2025 May 23;9:59. doi: 10.1186/s41927-025-00507-w (PMC12101040; doi:10.1186/s41927-025-00507-w)
Supplement: Supplementary file 2 — Additional file 2: Interview guide, description: The interview guide used for the qualitative phone interviews in German. [file 41927_2025_507_MOESM2_ESM.docx]

**Interview guide**

| Leitfrage/ Erzählimpuls | Checkaspekte | | Konkretisierende Fragen | | Aufrechterhaltungs- und Steuerungsfragen |
| --- | --- | --- | --- | --- | --- |
| Sie waren vor einiger Zeit im Universitäts- klinikum Erlangen und haben sich mit Verdacht auf Rheuma untersuchen lassen.  Könnten Sie mir bitte beschreiben, wann Ihre Symptome das erste Mal aufgetreten sind und was sie seitdem gemacht haben? | Warm-up  Patient-Journey  Weg durchs Gesundheitswesen  Hausarztpraxis  Facharztpraxis | | - Wann haben Sie das erste Mal gemerkt, dass etwas nicht stimmt?  - Wie haben Sie sich informiert?  - Mit wem haben Sie darüber gesprochen?  - Waren Sie in der Hausarztpraxis?  - Wie oft haben Sie in der letzten 7 Tagen ?! aus gesundheitlichen Gründen auf der Arbeit gefehlt? (Angabe in Stunden)    - Wie viele Stunden haben Sie in den letzten 7 Tagen tatsächlich gearbeitet?  - Wie stark haben sich Ihre gesundheitlichen Probleme in den letzten sieben Tagen auf Ihre Produktivität bei der Arbeit ausgewirkt?  0 (nicht) - 10 (sehr)  - Wie wichtig ist es Ihrer Meinung nach, frühzeitig zu erfahren, ob Sie eine Krankheit haben oder nicht?  1 (überhaupt nicht wichtig) - 10 (sehr wichtig)  - Welche Auswirkungen hat die frühzeitige Kenntnis Ihrer Diagnose auf Ihr tägliches Leben?  1 (überhaupt keine Auswirkungen) - 10 (sehr große Auswirkungen) | | Können Sie dazu noch mehr erzählen?  Und dann?  Wie war das für Sie?  Wie sehen Sie das?  Können Sie darauf bitte näher eingehen?  Könnten Sie dazu bitte ein Beispiel nennen?  Wie meinen Sie das konkret? |
| Bei Ihrem Besuch in der rheumatologischen Ambulanz im UK Erlangen haben Sie die Website *Rheumatic?* genutzt.  Bitte erinnern Sie sich. Wie kam es dazu? | Motivation  Erwartungen allgemein  Erwartete Veränderung  Eigene Einstellung | | - Wie wurden Sie über die Website informiert?  - Welche Erwartungen hatten Sie ursprünglich? Wie dachten Sie wird das?  - Hatten Sie auch Bedenken?  - Wie würden Sie Ihre Einstellung zu Digitalisierung oder Technik beschreiben?  (Sind Sie immer auf dem neuesten Stand? Oder eher nicht?)  - Wie leicht oder wie schwer fällt es Ihnen, sich in neue Technologien einzuarbeiten?  - Haben Sie schon einmal Technik oder Digitalisierung in Ihrer Gesundheitsversorgung genutzt? (in der Rheumatologie?) | | Können Sie dazu noch mehr erzählen?  Und dann?  Wie war das für Sie?  Wie sehen Sie das?  Können Sie darauf bitte näher eingehen?  Könnten Sie dazu bitte ein Beispiel nennen?  Wie meinen Sie das konkret?  Können Sie dazu noch mehr erzählen?  Und dann?  Wie war das für Sie?  Wie sehen Sie das?  Können Sie darauf bitte näher eingehen?  Könnten Sie dazu bitte ein Beispiel nennen?  Wie meinen Sie das konkret?  Können Sie dazu noch mehr erzählen?  Können Sie dazu noch mehr erzählen?  Und dann?  Wie war das für Sie?  Wie sehen Sie das?  Können Sie darauf bitte näher eingehen?  Könnten Sie dazu bitte ein Beispiel nennen?  Wie meinen Sie das konkret?  Können Sie dazu noch mehr erzählen? |
| Wie fanden Sie die Website? | Nutzer:innen-Erfahrung  Nutzungsverhalten  Benutzer:innen-Freundlichkeit  Handling  Visuelle, haptische Aspekte  Gegenüberstellung  Erwartungen vs. Realität  Aufwand | | - Wie lange hat das Ausfüllen gedauert?  - Haben Sie alles verstanden? Gab es Unklarheiten?  - Wussten Sie immer was Sie machen sollten?  - Was funktionierte gut?  - Gab es etwas, was nicht so gut funktioniert? Wenn ja, was?  - War die Website gut bedienbar?  - Was war das Ergebnis?  - Wenn Sie an Ihre ursprünglichen Erwartungen zurückdenken, entsprach die Website Ihren Erwartungen?  - Sehen Sie Risiken bei der Nutzung der Website?  - Gibt es etwas, was Sie an der Website verändern würden?  - War der Aufwand angemessen?  - Würden Sie die Website anderen Menschen mit Verdacht auf eine rheumatische Erkrankung weiter empfehlen? | |  |
| Wie verändert *Rheumatic?* Ihre rheumatologische Versorgung? | Versorgungsprozess  Ärzt:innengespräch  Persönliche Einstellung | | - Welchen Einfluss hat *Rheumatic?* auf das Arztgespräch bei der Rheumatologin / beim Rheumatologen?  - Sehen sich die Ärzt:innen das Ergebnis von *Rheumatic?* an? Gehen die Ärzt:innen darauf im Gespräch ein?  - Hat sich etwas für Sie verändert? Hatte der Fragebogen Einfluss darauf, wie Sie Ihre Symptome erleben?  Angenommen *Rheumatic?* wird zum Standard in der rheumatologischen Versorgung… also alle Menschen müssen zuerst den Fragebogen ausfüllen…   - Wie fänden Sie das? - Macht das die rheumatologische Versorgung besser? - Wer könnte den Fragebogen nutzen? Wer nicht? - Welche Barrieren sehen Sie?   Gibt es sonst noch etwas, was Sie sagen möchten? | |  |
| **Alter** | | **Geschlecht** | | **Aktuell: berentet/arbeitsunfähig/krankgeschrieben** | |
|  | |  | |  | |
| **Beruf** | | **Höchster Bildungsabschluss** | |  | |
|  | |  | |  | |
