# Supplementary material for: Rheumatic? A diagnostic decision support tool for individuals suspecting rheumatic diseases: Mixed-methods usability and acceptability study
Source: BMC Rheumatol. 2025 May 23;9:59. doi: 10.1186/s41927-025-00507-w (PMC12101040; doi:10.1186/s41927-025-00507-w)
Supplement: Supplementary file 3 — Additional file 3: Interview guide, description: The interview guide used for the qualitative phone interviews in English. [file 41927_2025_507_MOESM3_ESM.docx]

**Interview guide**

| Main question / narrative impulse | Checks | | Specific questions | | Maintenance and control questions |
| --- | --- | --- | --- | --- | --- |
| You were at the University Hospital Erlangen some time ago and were examined for suspected rheumatic disease.  "Could you please describe when your symptoms first appeared and what you have done since then?" | Warm-up  Patient-journey  Path through the healthcare system  General practitioner's office  Specialist practice | | - When did you first notice that something was wrong?  - How did you find information?  - Who did you talk to about it?  - Were you at the general practitioner's office?  - How many hours have you been absent from work in the last 7 days due to health reasons? (Indicate in hours)  - How many hours did you actually work in the last 7 days?  - How much have your health problems affected your productivity at work in the last seven days?  0 (not at all) - 10 (very much)  - In your opinion, how important is it to find out early if you have a disease or not?  1 (not at all important) - 10 (very important)  - What effects does early knowledge of your diagnosis have on your daily life?  1 (no effects) - 10 (very large effects) | | Can you tell me more about that? And then? What was that like for you? How do you see that? Can you please go into more detail? Could you give me an example? What exactly do you mean? |
| During your visit to the rheumatology clinic at the university hospital Erlangen, you used the website Rheumatic? Please remember. How did that happen? | Motivation  Expectations  Expected changes  Own attitude | | - How were you informed about the website?  - What were your original expectations? What did you think it  would be like?  - Did you also have any concerns?  - How would you describe your attitude towards digitalization  or technology? (Are you always up to date? Or not really?)  - How easy or difficult is it for you to learn new technologies?  - Have you ever used technology or digitalization in your  healthcare? (in rheumatology?) | | Can you tell me more about that? And then? What was that like for you? How do you see that? Can you please go into more detail? Could you give me an example? What exactly do you mean? |
| How did you find the website? | User experience  Usage behavior,  User-friendliness  Handling  Visual, haptic aspects Comparison  Expectations vs. reality  Effort | | - How long did it take to complete?  - Did you understand everything? Were there anything  unclear?  - Did you always know what you were supposed to do?  - What worked well?  - Was there anything that didn't work so well? If so, what?  - Was the website easy to use?  - What was the result?  - When you think back to your original expectations, did the  website meet your expectations?  - Do you see any risks in using the website?  - Is there anything you would change about the website?  - Was the effort reasonable?  - Would you recommend the website to other people with  suspected rheumatic disease? | |  |
| How does Rheumatic? change your rheumatological care? | Care process,  Doctor-patient interaction Personal attitude | | - What influence does Rheumatic? have on the doctor's appointment with the rheumatologist?  - Do the doctors look at the result from Rheumatic? Do the doctors address it in the discussion?  - Has anything changed for you? Did the questionnaire influence how you experience your symptoms?  - Assuming Rheumatic? becomes standard in rheumatological care... so everyone has to fill out the questionnaire first...  - How would you feel about that?  - Does that make rheumatological care better?  - Who could use the questionnaire? Who couldn't?  - What barriers do you see?  Is there anything else you would like to say? | |  |
| **Age** | | **Gender** | | **Current status:**  **retired/disabled/on sick leave** | |
|  | |  | |  | |
| **Profession** | | **Highest educational qualification** | |  | |
|  | |  | |  | |
